# Supplementary material for: Immunohistochemistry-based investigation of MYC, BCL2, and Ki-67 protein expression and their clinical impact in diffuse large B-cell lymphoma in upper Northern Thailand
Source: PLoS One. 2024 Jul 22;19(7):e0307253. doi: 10.1371/journal.pone.0307253 (PMC11262692; doi:10.1371/journal.pone.0307253)
Supplement: Attachment — Submitted filename: Response to editor and reviewer 14-6-67.docx [file pone.0307253.s002.docx]

Dear Editor:

We would like to express our gratitude to the reviewers for taking the time to read and provide valuable feedback like this. Below is our reply to your comment.

**The study group is small and diverse, collected from a single center without a correlation to the "gold standard" FISH. I think a broad revision could overcome this problematic errors. Please consider adding the nation of origin to the title and emphasizing the limited scope of the study in the introduction. Referring to the most recent version is necessary to indicate the study's restrictions at the time of publishing.**

- We considered adding the nation of origin to the title “Immunohistochemistry-based investigation of MYC, BCL2, and Ki-67 protein expression and their clinical impact in diffuse large B-cell lymphoma in Upper Northern Thailand”
- We Added a new paragraph regarding WHO-HAEM5 and the limited scope of the study in the introduction section. (page 5, lines 88-102)

**Reviewers:**

**Reviewer #2: The authors have made appropriate adjustments regarding the reviewer inputs. Minor details still need to be included. However, adding them depends on the journal's publication topic.**

**The WHO reference was still based on the revised 2016, while the new edition of WHO 2024 is already available.**

- Thank you for raising this issue. We have already added the paragraph that mentions the WHO-HAEM5 in the introduction section. (page 5, lines 88-102)

**Thank you for pointing out the RTU abbreviation, but the incubation details have not been included. Different heat and incubation duration options could be unique for various antibodies.**

- The incubation time was 16 minutes for MYC and 32 minutes for BCL2 and Ki-67. (page 8, lines 156-157)

**Thank you for the additional references for comparing the IHC technique with the others. However, the discussion has yet to include this study's limitation regarding IHC use, which might not always depict the gene rearrangement in the tumor cell.**

- We have added the limitation regarding IHC use in the discussion section (page 23, lines 400-401)

**Reviewer #3: I think that the paper does not contain relevant novel data that are woth publishing in a journal like PLOS. The clinical cohort is small, heterogeneous (mixture of all IPI groups), not from a registry or a trial but a single center collection. There is not even a correlation with the "golstandard" FISH, as far as I see. The issue of BCL2/MYC expression has been studied in much larger and more homogeneous cohorts and I simply do not understand what is added to the literature with this manuscript.**

- Thank you. We understand the issues you have highlighted. This study could not utilize the gold standard FISH technique due to limited resources and the heterogeneous cohort. However, our findings are significant for providing prognostic information. Double expressor can help classify disease prognosis in resource-limited settings. Although FISH is the gold standard according to WHO-HAEM5, the latest Thai Lymphoma Guideline 2022 stated that in cases where the FISH technique cannot be used, IHC detects MYC and BCL2 expression, referred to as double expression, which may correlate with a poorer prognosis than those without or with single expression. Our study demonstrates the value of the IHC technique in distinguishing different expression groups. This allows us to classify patients based on survival outcomes. Additionally, this finding can serve as basic information for Thai patients. We also recognize our study's limitations, which we have already discussed in the discussion section.
